# Comparative study on the mechanical mechanism of confined concrete supporting arches in underground engineering

**DOI:** 10.1371/journal.pone.0191935

**Published:** 2018-02-15

**Authors:** Zhijin Lv, Qian Qin, Bei Jiang, Yingcheng Luan, Hengchang Yu

**Affiliations:** 1 Taiyuan University of Technology, Taiyuan, China; 2 Erlintu Mining, Huineng Group, Neimenggu, China; 3 Shandong University, Jinan, China; Massachusetts Institute of Technology, UNITED STATES

## Abstract

In order to solve the supporting problem in underground engineering with high stress, square steel confined concrete (SQCC) supporting method is adopted to enhance the control on surrounding rocks, and the control effect is remarkable. The commonly used cross section shapes of confined concrete arch are square and circular. At present, designers have no consensus on which kind is more proper. To search for the answer, this paper makes an analysis on the mechanical properties of the two shapes of the cross-sections. A full-scale indoor comparative test was carried out on the commonly used straight-wall semi-circular SQCC arch and circular steel confined concrete arch (CCC arch). This test is based on self-developed full-scale test system for confined concrete arch. Our research, combining with the numerical analysis, shows: (1) SQCC arch is consistent with CCC arch in the deformation and failure mode. The largest damages parts are at the legs of both of them. (2) The SQCC arch’s bearing capability is 1286.9 kN, and the CCC arch’s ultimate bearing capability is 1072.4kN. Thus, the SQCC arch’s bearing capability is 1.2 times that of the CCC arch. (3) The arches are subjected to combined compression and bending, bending moment is the main reason for the arch failure. The section moment of inertia of SQCC arch is 1.26 times of that of CCC arch, and the former is better than the latter in bending performance. The ultimate bearing capacity is positively correlated with the size of the moment of inertia. Based on the above research, the engineering suggestions are as follows: (1) To improve the bearing capacity of the arch, the cross-sectional shape of the chamber should be optimized and the arch bearing mode changed accordingly. (2) The key damaged positions, such as the arch leg, should be reinforced, optimizing the state of force on the arch. SQCC arches should be used for supporting in underground engineering, which is under stronger influence of the bending moment and non-uniform load on the supporting arches. The research results could provide a theoretical basis for the design of confined concrete support in underground engineering.

## Introduction

**Fig 1:**
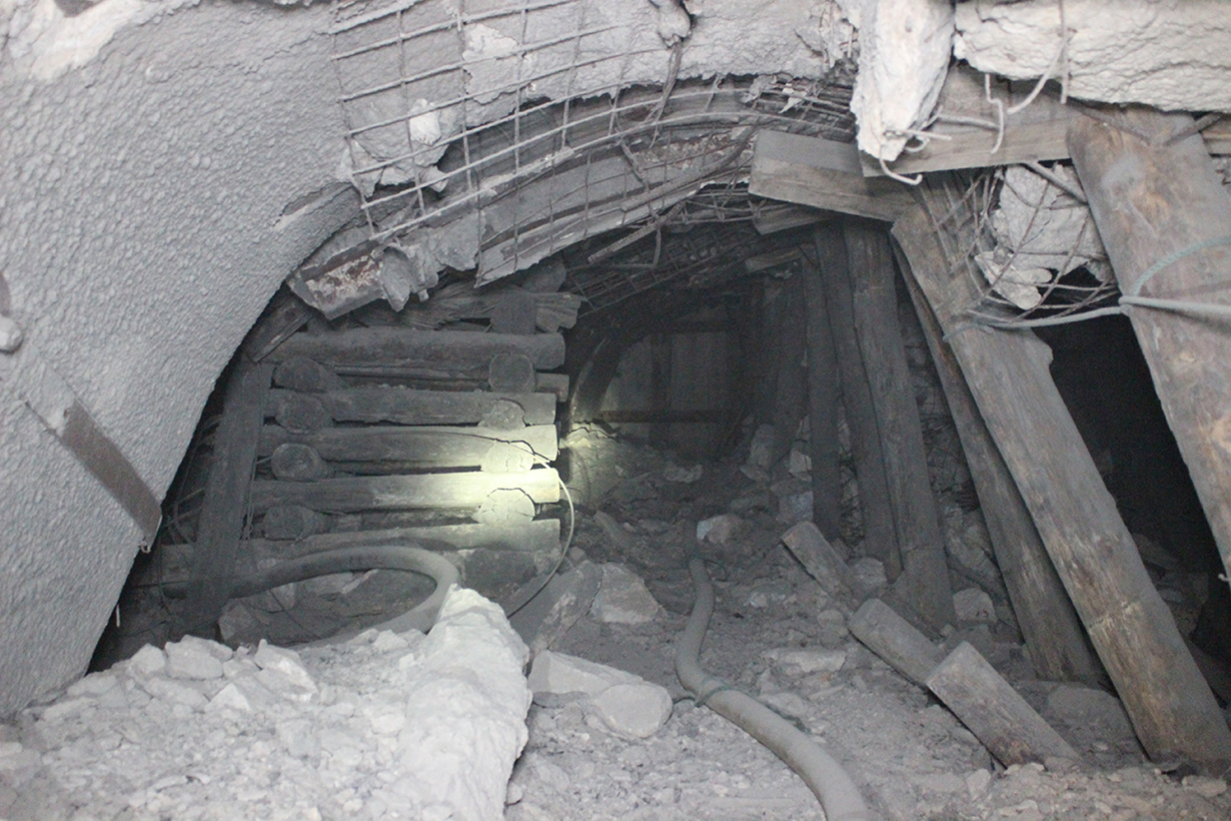
Damage of commonly used steel arch in underground engineering on the field.

In recent years, underground engineering is continuous to develop. So, high stress has become one of the key scientific problems of rock mechanics and engineering geology in engineering construction [1, 2, 3]. Many experts have carried out various researches on deformation control of surrounding rocks under high stress [4, 5, and 6]. The commonly used steel arch is insufficient in strength and often causes fracturing. And the instability of the arch leads to buckling and unstable failure, as shown in Fig 1. So the arch could not play its full bearing capacity [7, 8]. In addition, other scholars have discussed the bifurcation and stability behaviors in ground engineering [9].

Confined concrete component is made of steel with concrete inside, so that the two produce "force of symbiosis", and give full play to the advantages of both. The confined concrete structure has many advantages, such as high bearing capacity, good plasticity, convenience in construction and obvious economic, so it has been widely adopted in ground engineering [10, 11, 12, 13, 14, 15, 16, 17]. In recent years, researchers, including the authors’ team, have applied the confined concrete supporting method to underground engineering with complex conditions [18, 19].

Based on the commonly used U-shaped steel arch, Wang et al. [20] designed a confined concrete supporting structure. The open side of the U-shaped steel is closed by a sealing plate and the steel is filled with concrete. Its control effect is obviously superior to that of the conventional U-shaped steel arch. Wang et al. [21] proposed a new type of supporting method of square steel confined concrete (SQCC) for the deep underground roadway with fault fracture zones, and they conducted researches on the main supporting component—SQCC arch. Gao et al. [22] applied the circular steel confined concrete arch in the roadway with soft rocks and dynamic pressure, and they tested the mechanical properties of the concrete filled steel tubes and optimized the arch structure. These studies all show that the strength of confined concrete arch is 2 to 3 times higher than that of the conventional I-beam or U-shaped steel arch [23], and the confined concrete arch has good control effect in underground engineering applications.

The cross-section shape of the confined concrete arch is square or circular. At present, designers have no consensus on the cross-section shapes, so further study is necessary on the mechanical properties of the arch to make a reasonable choice on the cross-section shapes. For this purpose, the authors have conducted a comparative laboratory test on full-scale arches of both SQQC and CCC. The test is conducted with a self-developed full-scale test system for confined concrete arches. With the numerical calculation, studies are conducted on the ultimate bearing capacity and the deformation damage mechanism of the arch. Some suggestions are made on the arch shape selection.

## Engineering background and field application

### Engineering background

**Fig 2:**
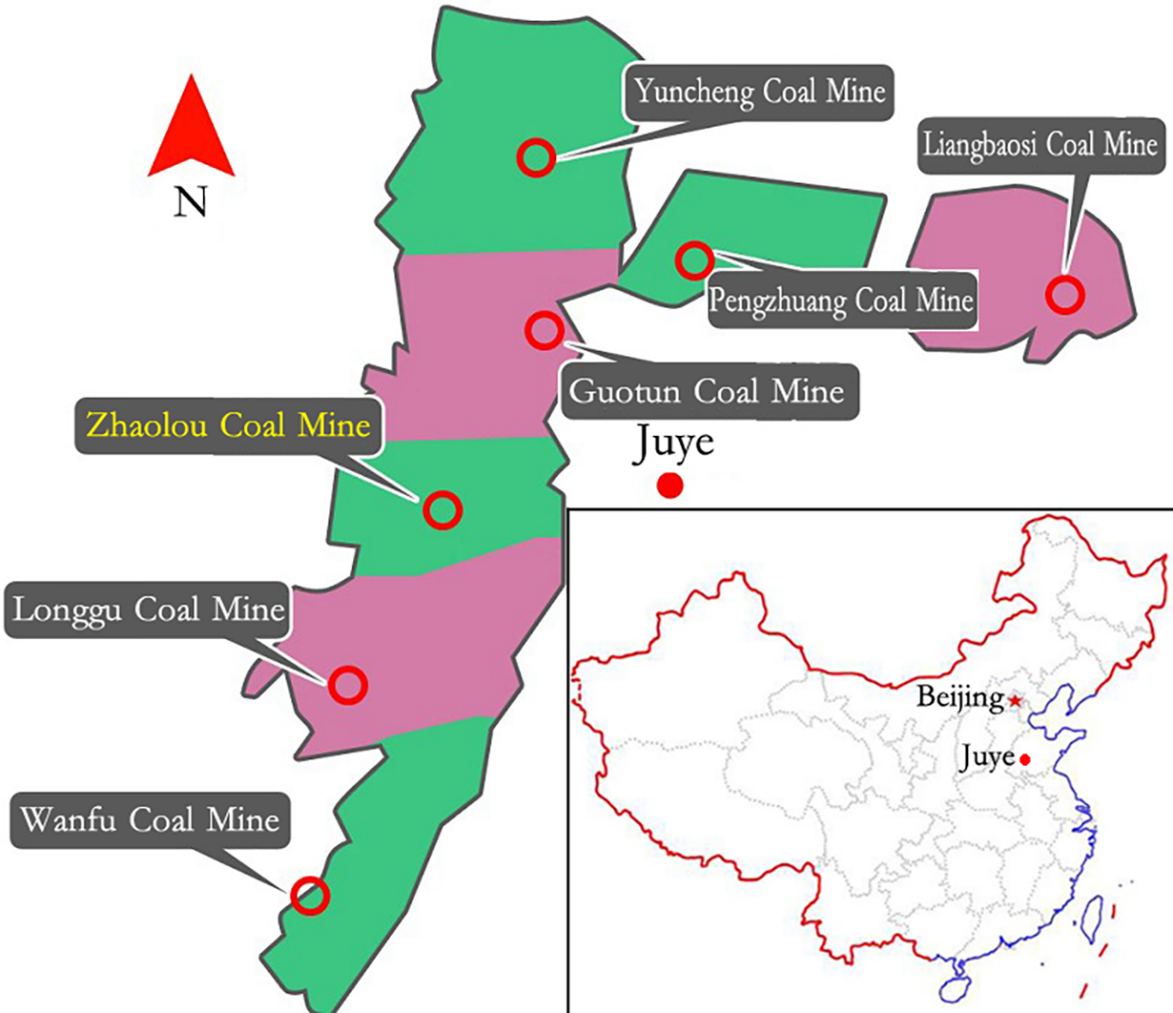
Geographical location of Zhao Lou Coal Mine.

At present, the geological conditions in the mine are the most complicated in underground engineering. In this paper, the Zhao Lou Coal Mine, which is located in the east of China, is selected for study as a typical example for this difficult underground support, as shown in Fig 2. With its maximum depth of 940.0m, Zhao Lou Coal Mine faces high stress problems in its roadways and difficulties in supporting. The shape of the roadway cross-section is straight-wall semicircle with net width of 5000mm and net height of 4300mm. The original support scheme is U29 steel arch with anchor bolt (anchor cable). Under the scheme, obvious and large deformation, concrete falling off, serious roof cracking, and arch breaking and buckling can be seen, as shown in Fig 1.

### Field applications

**Fig 3:**
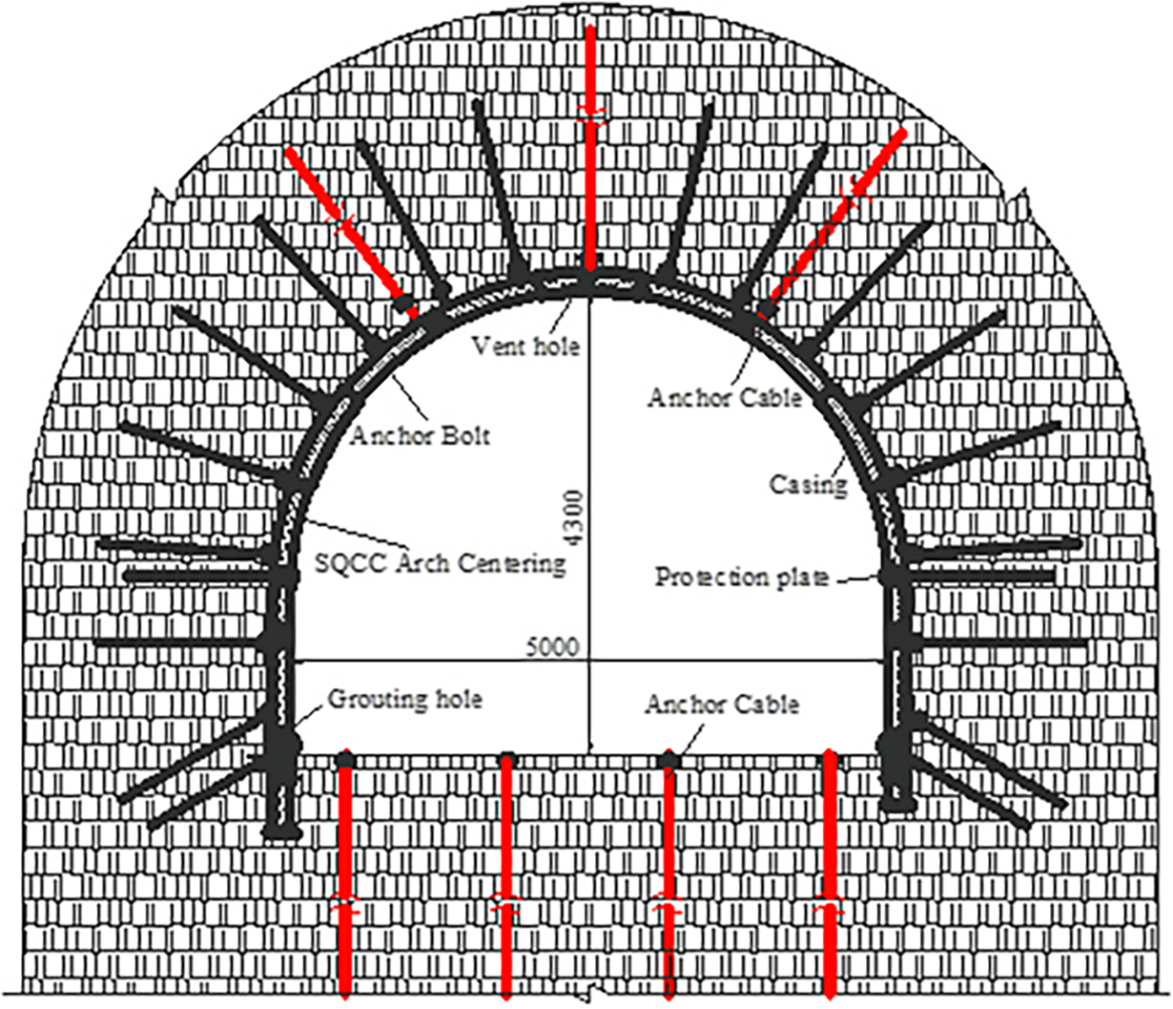
The cross-section layout of SQCC high-strength support system.

Based on the above engineering background, the field application of SQCC high-strength supporting method is carried out, which is discussed in another paper [21]. SQCC arch consists of steel pipes with section length of 150mm, wall thickness of 8mm and C40 concrete filled inside. The parameters of its bolt-mesh-spurting support are the same as those of the original support scheme (the cross-section layout is shown in Fig 3).

After implementing SQCC supporting method for a year, the monitoring results show that the maximum deformation of the arch is 27.7mm. Due to the high-strength support of the SQCC arch, the arch deformation is small, and the surrounding rock control effect is obvious.

The above studies show that SQCC has a good supporting effect on the deformation control of surrounding rock. Although some scholars have studied confined concrete arches in underground engineering [24, 25], their study was focused only on the confined concrete arch with steel pipes of circular cross-sections. At present, the designers have no consensus on arch cross-section selection. In order to clarify the advantages and the application range of the arches with different cross-sections, the authors have conducted a full-scale laboratory comparison experiment. The experiment is full-scale on arches with circular and square cross-section.

## Methods

### Laboratory test

#### Full-scale test system for confined concrete arches

The present devices cannot perform 1:1 scale laboratory test on arches due to its insufficient strength and limited size, which could result in deviation between test result and real data. Therefore, the authors developed a new type of full-scale test system for confined concrete arches.

**Fig 4:**
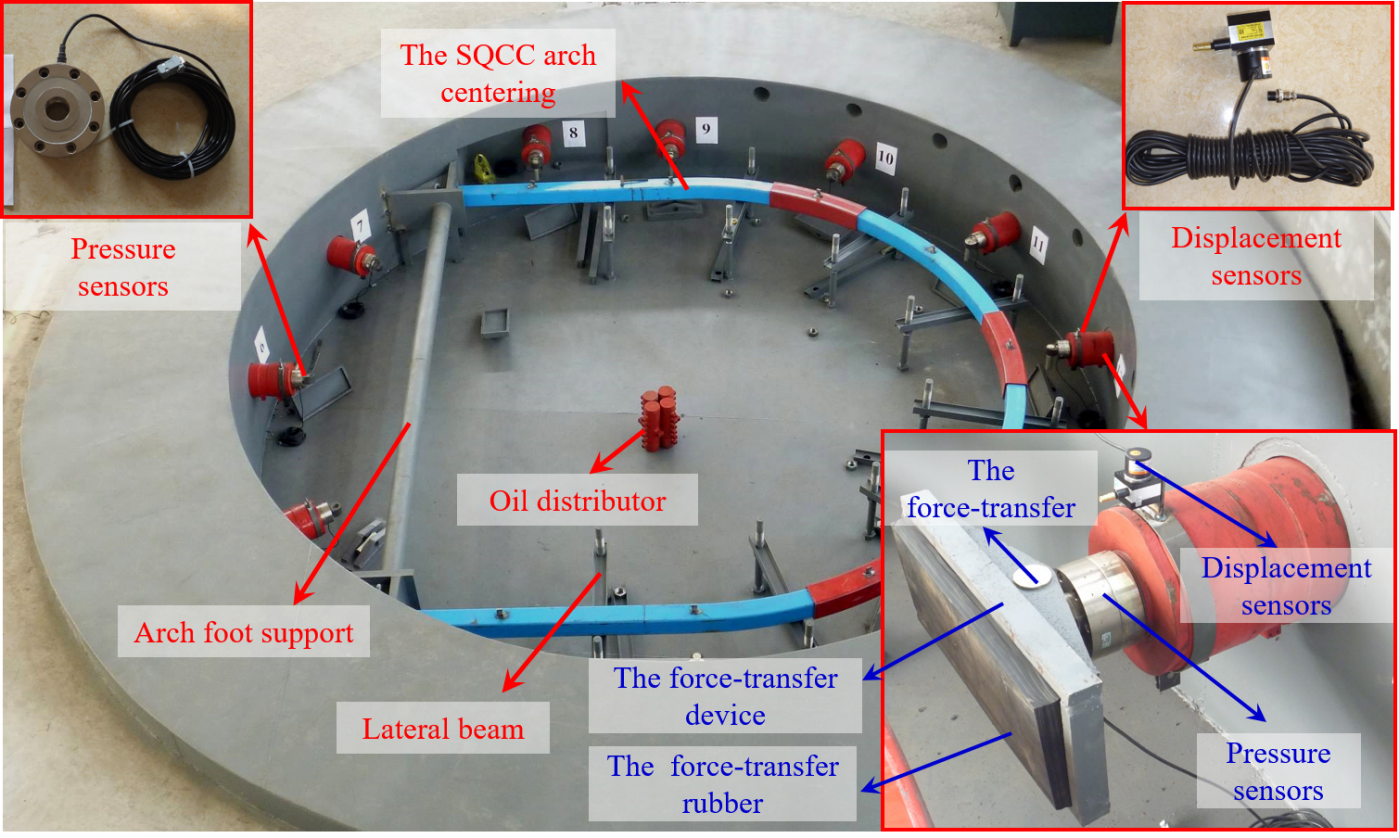
Confined concrete arch full-scale test system.

The test system consists of reaction devices, loading and controlling devices, monitoring devices and its ancillary components, as shown in Fig 4.

With an outer diameter of 10m, the reaction device is ladle concrete structure. It can provide more than 2400t reaction force with many advantages such as large size, high strength and stiffness. The loading and controlling devices consist of a hydraulic pump station, hydraulic cylinders, automatic measurement and control devices, etc. They can achieve synchronized loading on 12 cylinders in two groups. The monitoring devices consist of monitors of radial force, radial displacement, angle, stress and deformation.

#### Arch laboratory test plan

**Fig 5:**
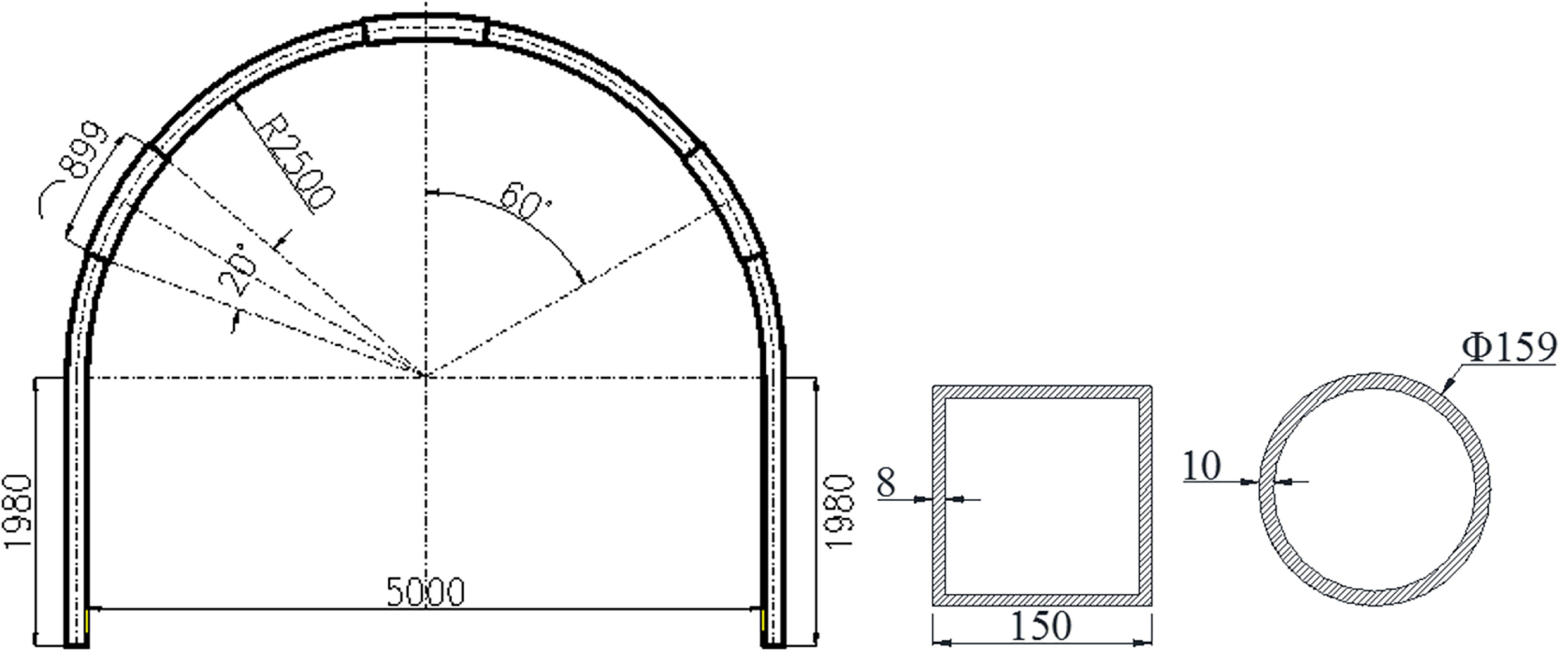
Arch centering dimension and section form. (a) Arch dimension. (b) Arch section form.

**Fig 6:**
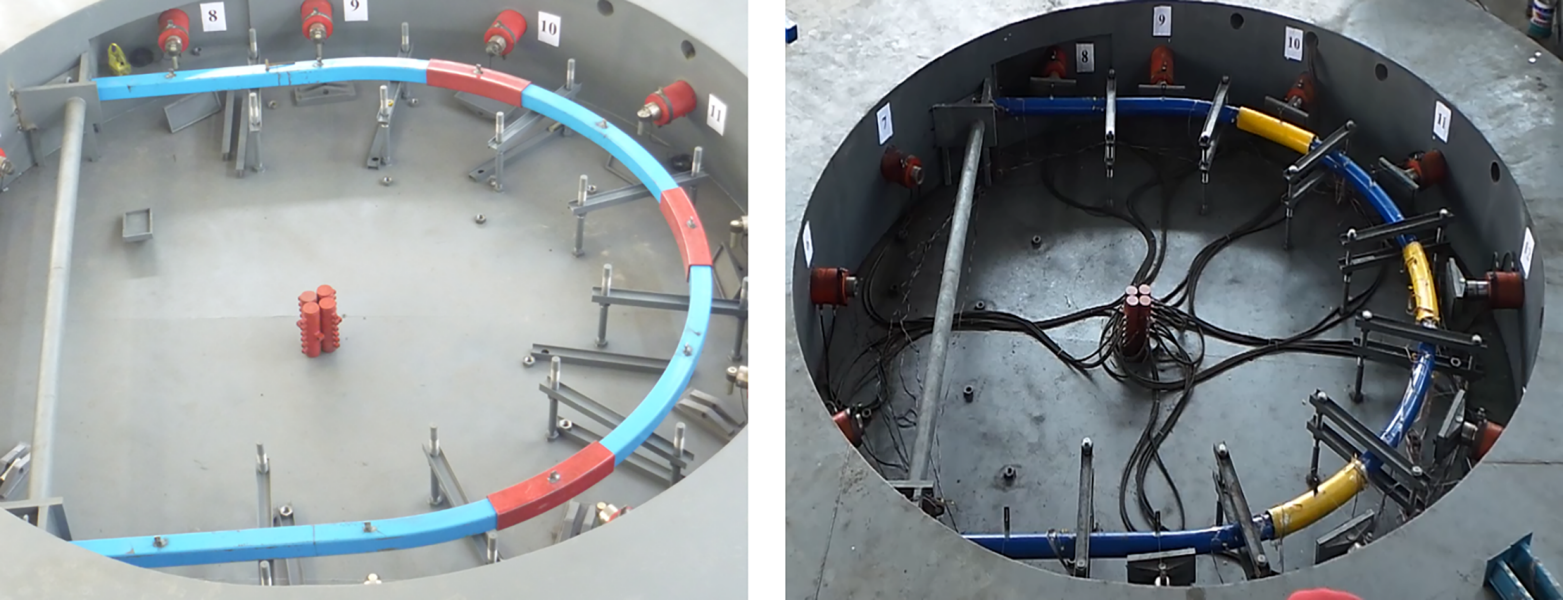
Arch centering test. (a) SQCC arch test. (b) CCC arch test.

Arch test: to compare accurately the arch mechanical properties and reflect the real field situation, this experiment is designed for cross-sections of SQCC and CCC arches in full-scale. The two kinds of arches have the same steel content. The SQCC arch is made of square steel pipes with the cross-section length of 150mm and the wall thickness of 8mm. The CCC arch is made of circular steel pipes with the cross-section diameter of 159mm and wall thickness of 10mm. All the arches are filled with C40 concrete. The cross-sectional area of SQCC arch steel pipe is 4544mm^2^ and the area of CCC arch steel pipe is 4681mm^2^, and the difference ratio between the two is 3.01%. The specific dimensions of the test arch are shown in Fig 5. The SQCC arch and CCC arch are shown in Fig 6 before the start of the test.

**Fig 7:**
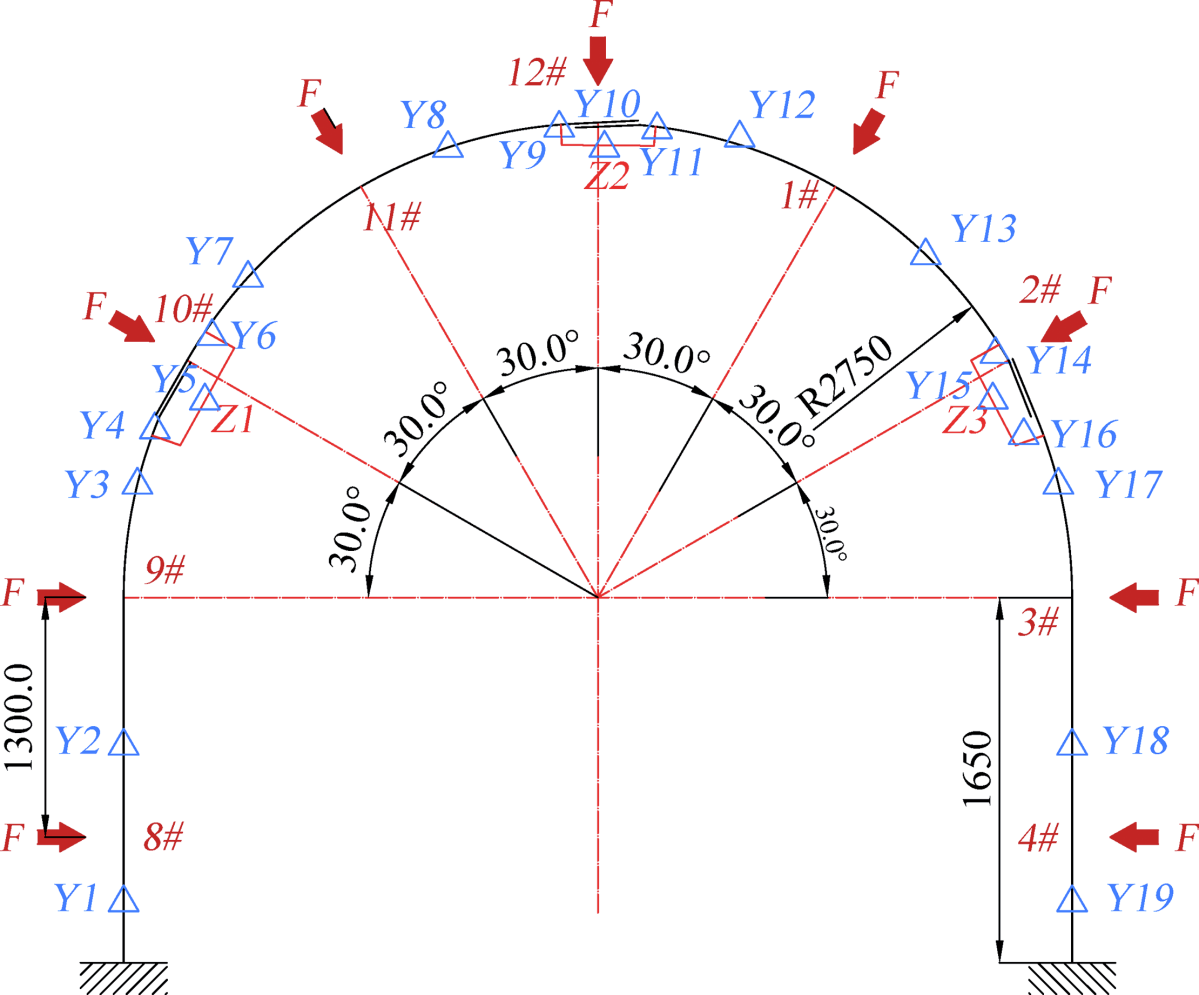
The layout of the key monitoring points for monitoring the force and deformation in the arch test process.

Arch monitoring plan: the monitoring test is conducted according to layout of the monitoring points shown in Fig 7 and the specific monitoring item statistic is listed in Table 1. Analysis is made on the force and the deformation in the arch test process.

**Table 1:** The table of monitoring statistics.

Monitoring content	Sensor/type	Quantity	Sample frequency	Number
**Radial force**	Wheel spoke force sensor/60t	9	1s	1#~4# 8#~12#
**Radial displacement**	Pull line displacement sensor/1000mm	9	1s	1#~4# 8#~12#
**Steel strain**	strain gage	13 monitoring points/ 33 pieces	2s	Y1~Y19

Loading method: test with uniform loading.

Loading rate and holding time: graded loading is adopted. As the load is less than 90% of the expected ultimate load, the loading rate is 10kN/min with a holding time of 0.5min for every 30kN. As the load is greater than 90% of the expected ultimate load, the loading rate is 5kN/min with a holding time of 0.5min for every 10kN.

Loading stop criteria: a monotonically pressurized way is adopted for loading. During the test, the damage of the specimen is observed, and loading is stopped when the entire specimen enters the yield state or are significantly damaged.

### Numerical test

The numerical calculation software ABAQUS is used to study confined concrete arches. It is for validating the experimental results with the laboratory test of the arch and obtaining the test data which cannot be effectively collected in the arch test.

#### Numerical test material constitutive relationship

The constitutive relationship of steel and core concrete is based on the results of reference [26], and the stress-strain relationship is adopted which is applied to ABAQUS.

#### Establishment of numerical experiment model

**Fig 8:**
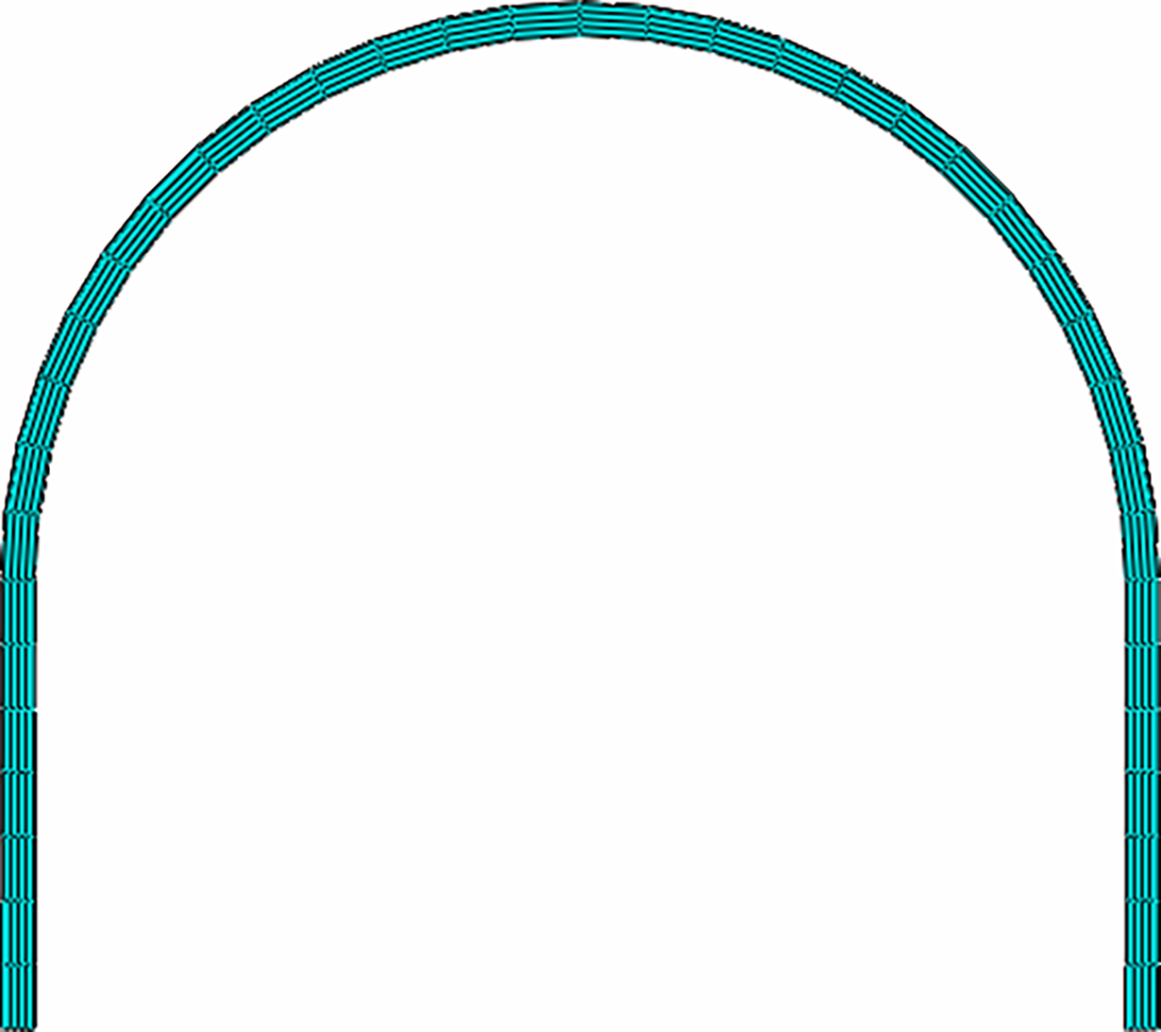
The numerical model establishment and meshing.

**Fig 9:**
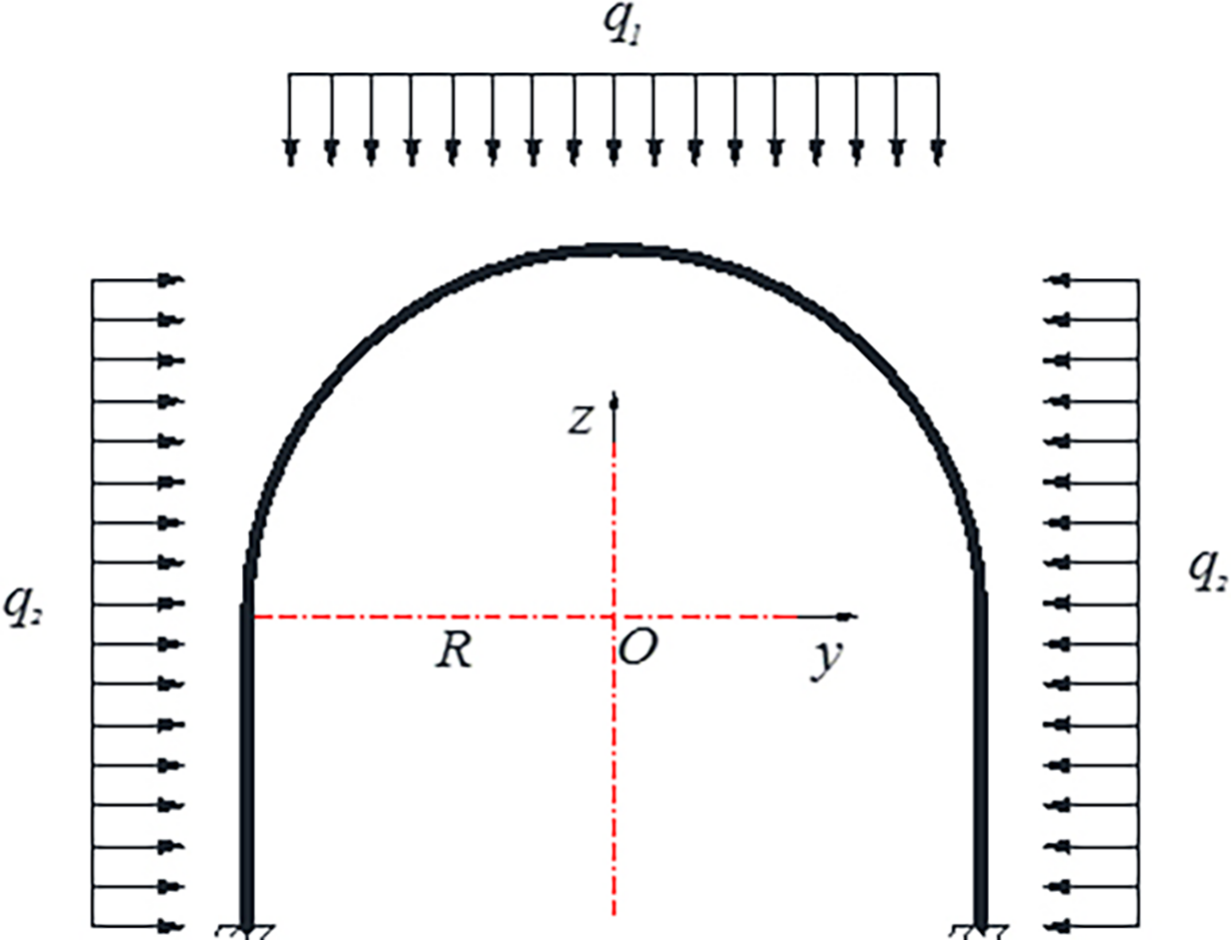
Loading schematic diagram of the arch at numerical experiment.

3D solid element is adopted for steel pipe and core concrete and C3D8R is selected for element type. Assume that the overall deformation of steel and concrete is compatible and there is a tie constraint between the two. Loading is carried out with surface load being applied on the lateral surface of the arch. The model establishment and meshing are shown in Fig 8, and the arch load application method is shown in Fig 9.

## Results and discussion

### Comparative analysis on mechanical properties of arch

#### Analysis on arch load curve

**Fig 10:**
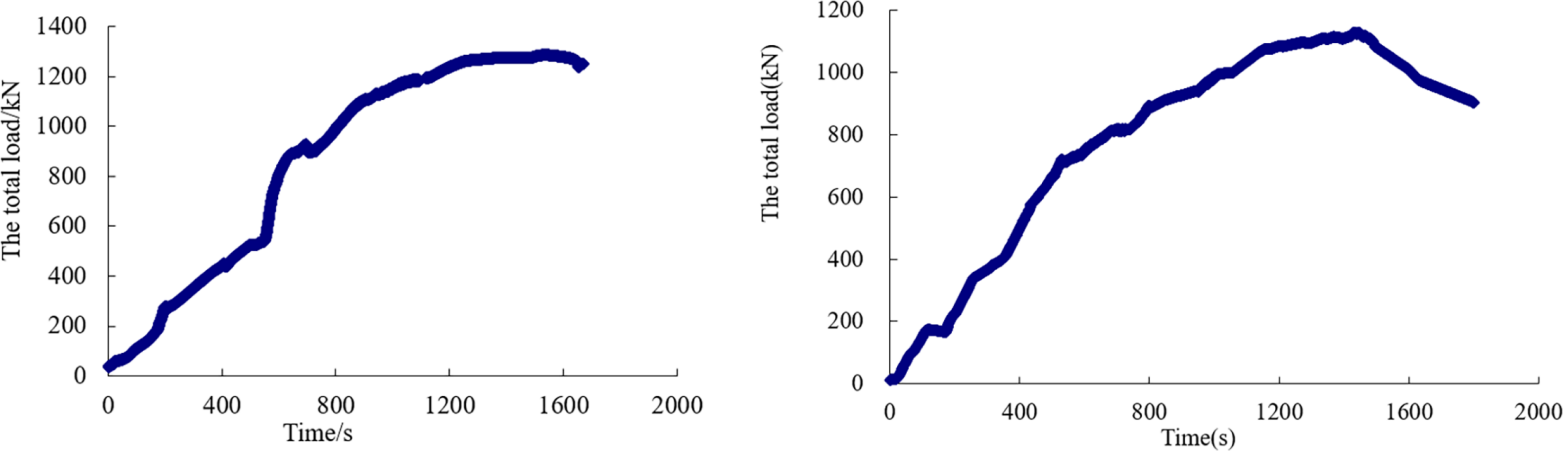
Curve of the arch specimen’s overall load vs. time. (a) Curve of the SQCC arch’s overall load vs. time. (b) Curve of the CCC arch’s overall load vs. time.

**Fig 11:**
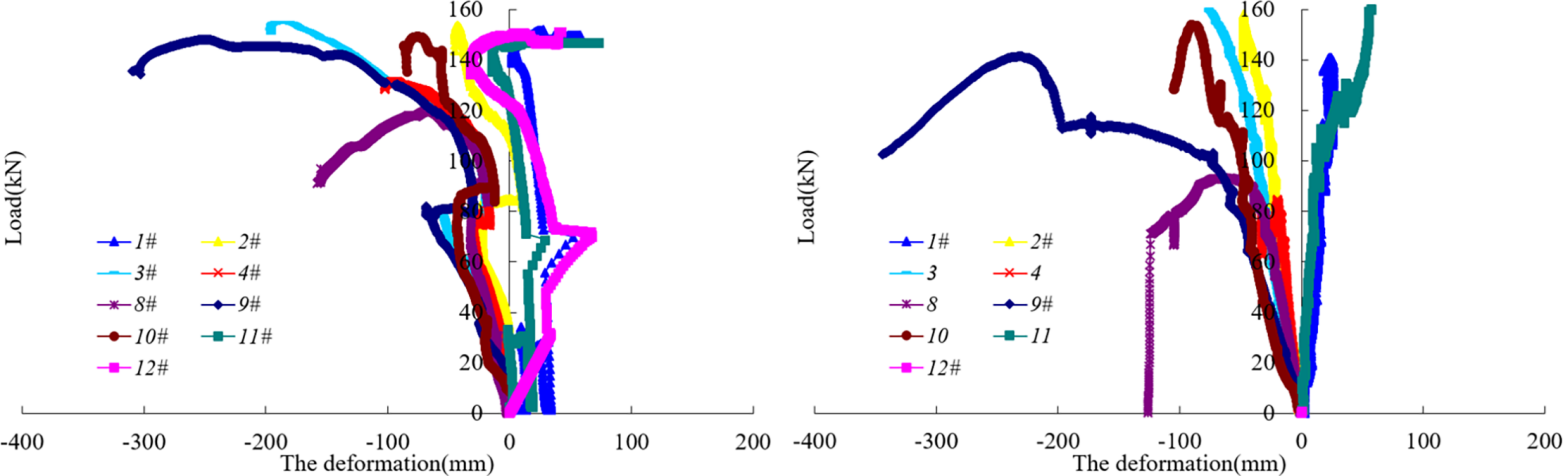
Curve of individual cylinder load vs. displacement. (a) Curve of SQCC arch’s individual cylinder load vs. displacement. (b) Curve of CCC arch’s individual cylinder load vs. displacement.

Fig 10 shows the variation curve of the total load of the 9 groups of cylinders over time in the SQCC and CCC arch laboratory tests. Fig 11 shows the radial load-displacement curve for an individual cylinder in the SQCC and CCC arch tests.

From Fig 10 and Fig 11, the following can be analyzed:

1. The SQCC arch’s bearing capability is 1286.9 kN, and the CCC arch’s ultimate bearing capability is 1072.4kN. Thus, the SQCC arch’s bearing capability is 1.2 times that of the CCC arch. With a larger bearing capacity, SQCC arch can provide a stronger radial force for the surrounding rock, and it also can effectively maintain the stability of the surrounding rock and facilitate the self-bearing capacity of the surrounding rock.
2. The radial load displacement curve shows SQCC and CCC arches have negative displacement at 2#, 3#, 4#, 8#, 9# and 10# points, indicating a retracted deformation trend. The ultimate bearing capacity of the SQCC arch is 1248kN in numerical calculation and 1286.9kN in the full-scale laboratory test, and the difference ratio between the two is 3.02%. The ultimate bearing capacity of the CCC arch is 1102.6kN in numerical calculation and 1072.4kN in the laboratory test, and the difference ratio is 2.82% between the two. The good consistency between the two verifies the correctness of the numerical calculation.
3. As the test continues, the arch has reached the ultimate bearing capacity, and the radial displacement at the 9# monitoring point on CCC arch continues to increase. CCC arch has a larger displacement value than SQCC arch does in the similar positions. The deformation is serious, and the damage occurs at its leg, which indicates SQCC arch is more effective in controlling large deformation of surrounding rock.

#### Arch deformation and failure phenomenon

**Fig 12:**
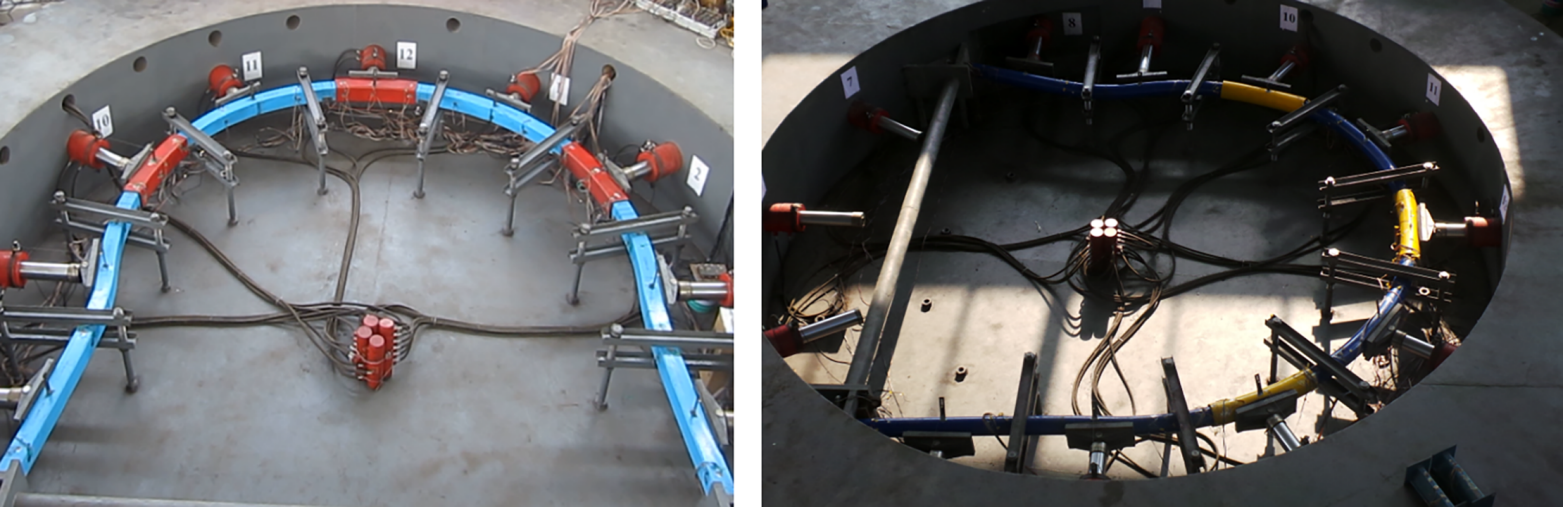
Arch specimen’s failure shape. (a) Final SQCC arch failure shape. (b) Final CCC arch failure shape.

Comparison of the overall deformation and failure between SQCC and CCC arches:

The test of SQCC and CCC arches lasts about 30 minutes and can be divided into four stages. The first stage: No significant deformation of the arch is observed for a long period after the test begins and the forward thrust from all cylinders uniformly increases. The second stage: As the load continues to rise, the overall shape of the arch becomes thinner and the left and right sides are squeezed inward. In about 10min, CCC arch has a more obvious bending deformation at its leg than SQCC arch does. The third stage: In about 15min, the arch leg continues to squeeze inward and both SQCC and CCC arches enter the yield state. At this moment, the total load of the SQCC arch is about 1100 kN and the CCC arch is about 950 kN. The fourth stage: In about 30min, the cylinder load has reached the maximum value and SQCC and CCC arches have reached their maximum deformation. The largest deformation is on the arch legs. The final failure shape of the arch was shown in Fig 12.

**Fig 13:**
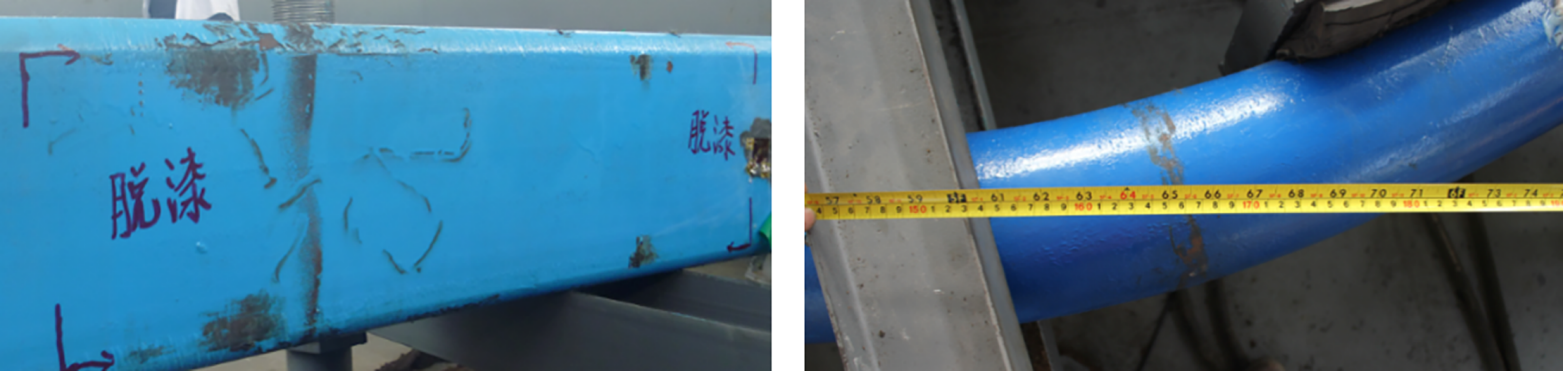
Arch leg strength failure mode. (a) SQCC arch leg failure. (b) Maximum deformation in CCC arch leg on the position of 1.55m-1.75m high.

The analysis on the key deformation and failure positions of SQCC and CCC arches shows:

1. The paint skin is peeled off 1.0–2.0m high on the legs of SQCC arch. The phenomenon indicates that steel in the position has reached the plastic state, but the steel has not yet been obviously damaged, and the cross-section shape of the square steel still maintains unchanged, as shown in Fig 13(A). It is also indicated that the arch leg can still withstand a certain load and does not produce obvious damage to the overall arch.
2. The confined steel pipe is bent and deformed in the left and right sides of CCC arch legs, and the damages are 1.55m-1.75m high on the legs and basically symmetrical at both left and right sides as shown in Fig 13(B). At the same time, the paint skin is obviously peeled off at its legs, indicating that the steel in the position has reached the plastic state. The circular cross-section basically maintains unchanged, indicating that CCC arch can still continue to bear certain load.

#### Internal force analysis of the arch

**Fig 14:**
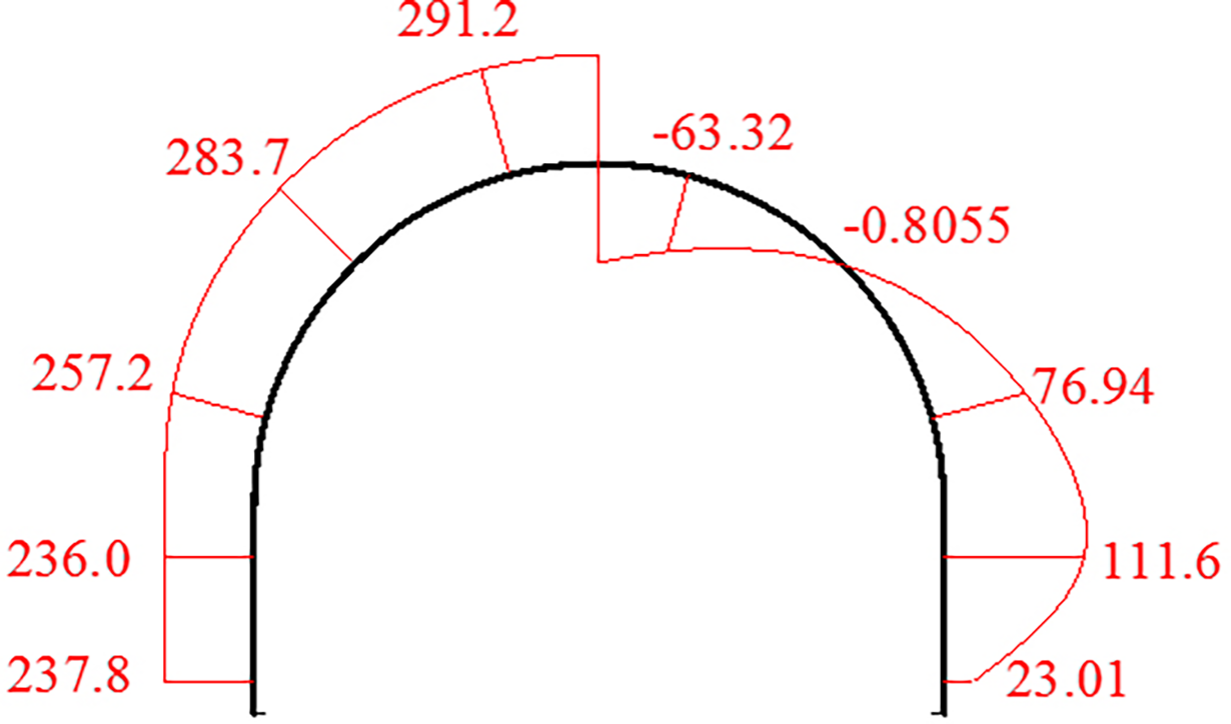
Diagram of the SQCC arch’s internal force. Left side is axial force/kN, Right is bending moment/kN·m.

The internal force of the arch is extracted from the corresponding position of the SQCC arch cross sections in the numerical test to monitoring positions shown in Fig 7. Table 2 shows the axial force and bending moment of the typical section when the arch reaches the ultimate bearing capacity, and the values are drawn at left side and right side respectively in Fig 14.

**Table 2:** Axial force and bending moment at the measurement point.

	Y1	Y2	Y3	Y7	Y8
**Axial force/kN**	237.8	236.0	257.2	283.7	291.2
**Bending moment/kN·m**	23.0	111.6	76.94	-0.81	-63.3

As shown in Fig 14, the SQCC arch is subjected to uniform load, and the axial force gradually increases from the skewback to the vault, but the overall difference is not large. The maximum axial force is located on the arch vault with a value of 291.2kN. According to the reference [23], this value is only 12% of the ultimate load of SQCC short column components under axial compression. The maximum bending moment of the arch is 111.6kN·m, 1.0 to 2.0m high on the arch legs. The bending moment is positive, and the arch leg is bent to make the arch legs squeezed inside. The failure mode is consistent with that from the laboratory test. The above shows that the arch damage is mainly caused by the bending moment. Therefore, based on the engineering practice, the method should be adopted to optimize the cross-section shape of the chamber and to change the arch bearing mode. It is also for adjusting the internal force distribution of the arch, and improving the bearing capacity of the arch and the self-bearing capacity of the surrounding rock.

#### Discussion on the influencing factors of mechanical properties of arches

A numerical calculation is conducted on SQCC and CCC arches with the same steel content. It is to compare and analyze the influence of different factors on the mechanical behavior of the confined concrete arches under the influence s of different pipe wall thicknesses and different core concrete strength.

Analysis on wall thickness effects:

In order to compare and analyze the influence law of different steel tube thickness on the bearing capacity of the arch, SQCC arches are selected with steel tube thickness of 4mm, 6mm, 8mm, 10mm and 12mm. CCC arches are selected with steel tube thickness of 5mm, 7mm, 10mm, 12mm and 14mm. Five sets of comparative numerical calculation schemes are designed. The serial numbers are B1-B5 and the specific schemes are shown in the Table 3. Under each scheme, the steel content of SQCC and CCC arches basically is the same, and the core concrete is made of C40.

**Table 3:** Numerical calculation schemes of different steel tube thicknesses of SQCC and CCC with the same steel content.

Number	Tube models	Area/mm^2^	Different rate
**B1**	C159×5	2419	3.43%
	SQ150×4	2336	
**B2**	C159×7	3343	3.27%
	SQ150×6	3456	
**B3**	C159×10	4681	3.01%
	SQ150×8	4544	
**B4**	C159×12	5542	1.04%
	SQ150×10	5600	
**B5**	C159×14	6377	3.73%
	SQ150×12	6624	

**Fig 15:**
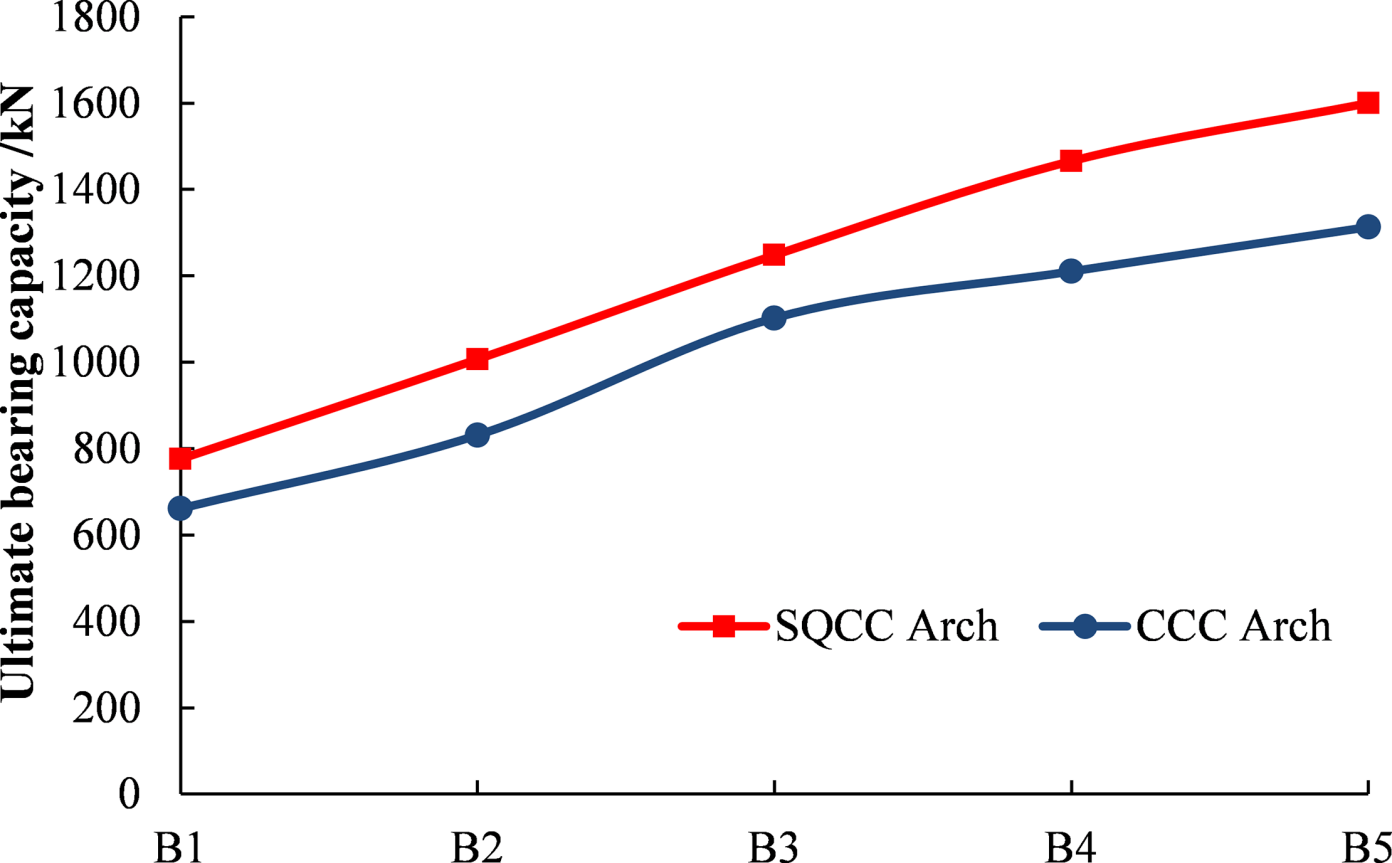
The influence curve of steel tube thickness on the ultimate bearing capacity of the confined concrete arch.

Through numerical calculation and analysis of the above scheme, the influence curve of the wall thickness on the ultimate bearing capacity of the confined concrete arch is obtained, as shown in Fig 15.

From Fig 15, the following can be analyzed: ① The ultimate bearing capacity of SQCC and CCC arches increases with the increase of wall thickness. ② The ultimate bearing capacity of SQCC arch is higher under the condition of the same steel content in arch cross-section.

Analysis on the impact of core concrete strength:

In order to study the influence of core concrete strength on the bearing capacity of the arch, the types of concrete are selected with different strength. The type numbers are C30, C40, C50, C60, C70 and C80. Six numerical calculation schemes are designed. The dimensions of SQCC arch and CCC arch are 150×8mm and 159×10mm respectively. The mechanical parameters of steel pipe and concrete are selected as the above selected.

**Fig 16:**
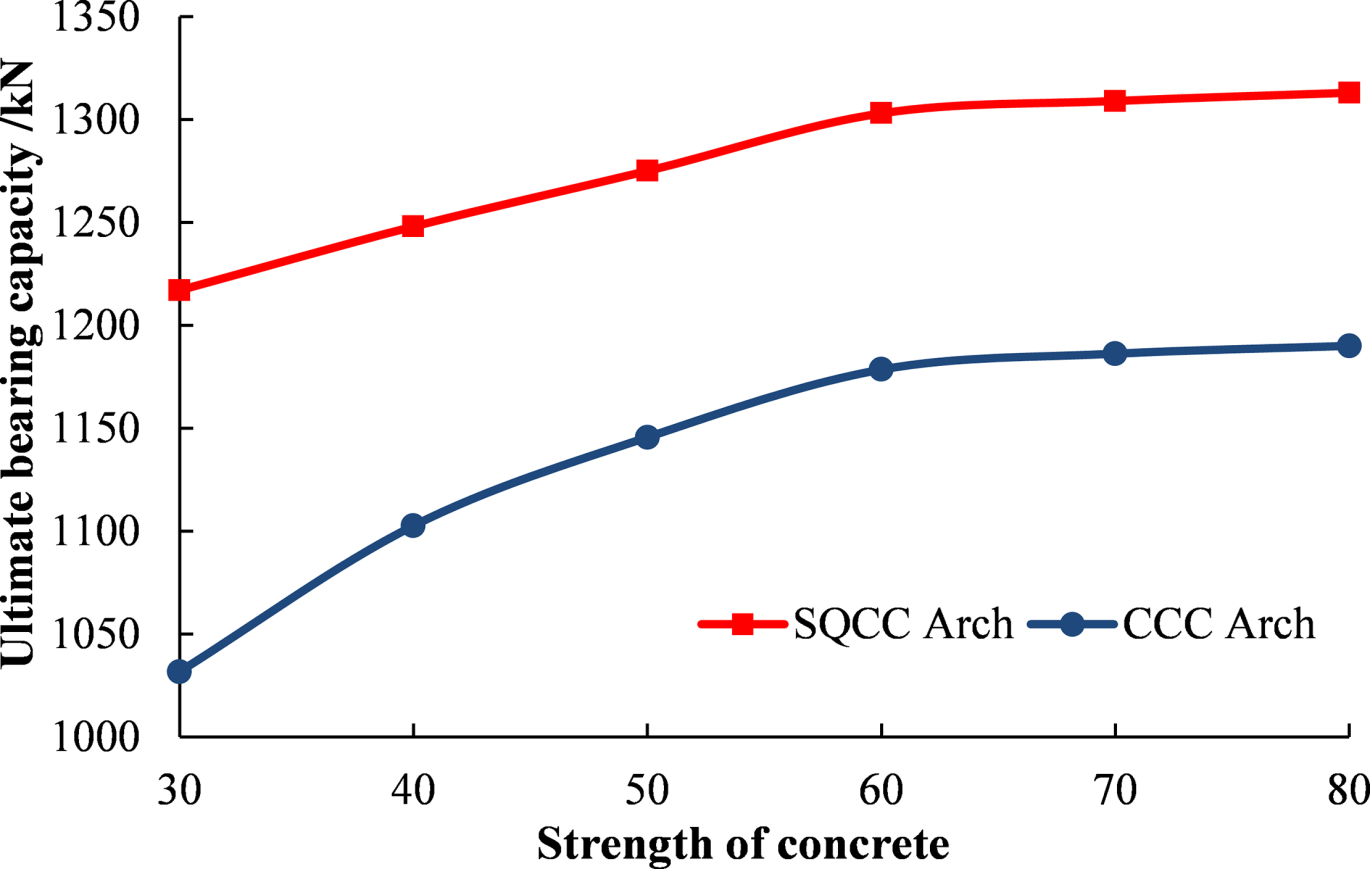
Influence curve of core concrete strength on ultimate capacity of confined concrete arch.

Through the numerical calculation and analysis of the above scheme, the influence curve of the core concrete strength on the ultimate bearing capacity of the confined concrete arch is obtained, as shown in the Fig 16.

From Fig 16, the following can be analyzed: ①The ultimate bearing capacity of the arches gradually increases with the increase of the core concrete strength. ②Under the same concrete strength, the ultimate bearing capacity of the SQCC arches is higher.

### Test summary and engineering proposal

1. The full-scale test of the straight-wall semi-circular arch commonly used in roadway indicates that the ultimate bearing capacity of the SQCC arch is 1286.9kN, which is 1.2 times of that of the CCC arch. So SQCC arch is more conducive to play the self-bearing capacity of the surrounding rock with a stronger radial support on the surrounding rock.
2. SQCC and CCC arches basically have the same failure mode, and the key failure positions are on their legs. In comparison, the deformation and bending are more obvious on the steel pipe of CCC arch, and SQCC arch has no obvious failure even in the case of large deformation. In the underground engineering support, the straight-wall semi-circular arch is subjected to combined compression and bending, is mainly affected by the bending moment damage. SQCC arch has obvious supporting effect. It is recommended to use the SQCC arch which has higher bending performance.
3. The numerical analysis on the arches with different core concrete strength and steel tube thickness shows the bearing capacity of SQCC and CCC arches increases with the increase of steel tube thickness and core concrete strength. Under the same conditions, the bearing capacity of SQCC arch is higher.
4. In the field application, the cross-sectional shape of the chamber should be optimized, guard plates should be welded in the critical damage positions on the arch legs, and the length of the arch leg should be reduced.

## Conclusion

1. In view of the problem of underground engineering support with high stress, the high-strength support system of SQCC is adopted to reinforce the control on the surrounding rocks, and good effect is achieved.
2. The full-scale test of the straight-wall semi-circular arch indicates commonly used in roadway that the failure modes of SQCC and CCC arches are basically the same, and the ultimate bearing capacity of SQCC arch is 1.2 times of that of CCC arch.
3. The numerical analysis on the arches with different core concrete strength and steel tube thickness shows the bearing capacity of SQCC and CCC arches increases with the increase of steel tube thickness and core concrete strength. Under the same conditions, the bearing capacity of SQCC arch is higher.
4. SQCC arch has obvious advantages in the underground engineering. Comparing with CCC arch, it has a better bending behavior. According to the actual engineering, SQCC arch is recommended for straight-wall semi-circular chamber with high stress.
